# Inhibition of S6K by resveratrol: In search of the purpose

**DOI:** 10.18632/aging.100059

**Published:** 2009-06-29

**Authors:** Mikhail V. Blagosklonny

**Affiliations:** Department of Cell Stress Biology, Roswell Park Cancer Institute, Buffalo, NY 14263, USA

**Keywords:** Aging, chaperone, growth hormone, longevity, Snell, stress

## Mechanisms of the anti-aging effects of resveratrol

Resveratrol, a natural agent found in grape skins, has been proposed to account for the beneficial effects of red wine against heart disease. Resveratrol prevents age-related diseases and extends lifespan in several species [1-9]. In this issue of Aging, Armour *et al* demonstrate that resveratrol directly inhibits S6 kinase (S6K) [10].

S6K is a downstream target of mTOR (mammalian Target of Rapamycin). In response to nutrients, growth factors and hormones, the mTOR/S6K pathway promotes cellular mass growth, which is needed for cell proliferation. In post-mitotic (non-proliferating) cells, mTOR promotes cellular senescence [11]. Rapamycin decelerates mammalian cell senescence [12]. Dephosphorylation of S6 is correlated with suppression of cellular aging by several agents [13-15]. Furthermore, inhibition of the TOR/S6K extends life span in yeast, worms and *Drosophila* [16-21]. Also, mice deficient for mTORC1 or S6K are protected against obesity [22,23]. Therefore, the discovery made by the David Sinclair laboratory [10] that resveratrol directly inhibits S6K is very important.

**Figure 1.**
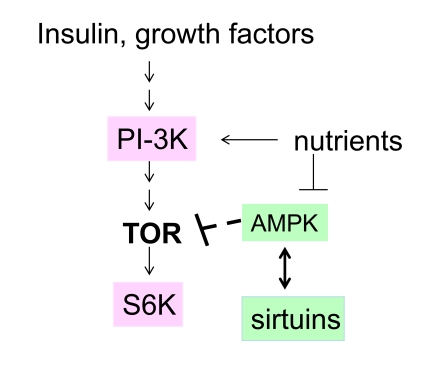
The nutrient-sensing pathway as a target of resveratrol. In red, targets that are inhibited by resveratrol. In green, targets that are activated by resveratrol.

In addition, resveratrol activates sirtuins [2,24-25] and AMPK [6,26], which may antagonize the TOR pathway downstream and upstream, respectively (Figure 1). It has been suggested that sirtuins and mTOR may be involved in the same longevity pathway [27]. By inhibiting S6K and activating sirtuins, resverat- rol may exert its anti-aging effect. But why would plants produce an anti-aging drug?

## Purpose of anti-aging effects of resveratrol

As suggested by David Sinclair and co-authors in their elegant xenohormesis hypothesis, by sensingresveratrol, animals can monitor their environment [28,29]. Resveratrol is produced by plants in response to stress. According to the 'xenohormesis hypothesis', organisms have evolved to respond to stress signaling molecules produced by other species. In this way, organisms can prepare in advance for a deteriorating environment and loss of food supply [28,29].

The discovery that resveratrol inhibits S6K further supports this hypothesis, because S6K is a part of the nutrient-sensing pathway. In other words, organisms ‘sense' the lack of nutrients before there is an actual decline in nutrients.

Here I propose an alternative (but not mutually exclusive) model, suggesting that anti-aging effects of resveratrol are ‘side effects' of its cytostatic effect. Given that the nutrient-sensing TOR/S6K pathway promotes cellular growth, plants produce resveratrol to inhibit fungal growth, thus protecting the grape. But the same S6K that drives growth also drives aging. In a recent issue of *Aging*, Michael Hall and I discussed how growth and aging are hardwired that suppression of growth also suppresses aging [30]. By inhibiting S6K, resveratrol should slow down both growth and aging.  Then "the purpose of resveratrol" is to inhibit fungal growth but its ‘side effect' is suppression of aging in a variety of species.

## Using toxins for different purpose

In general, living beings have created numerous "medical drugs" for a simple purpose: to harm other organisms, to protect themselves from predators, and to kill competitors. Plants, microorganisms and sea animals produce toxic agents that inhibit or damage microtubules, DNA, heat-shock proteins, deacetylases and the proteasome, for example. These cytotoxic agents are used as anti-cancer drugs, although nature did not create them for that purpose. Some agents can hurt organisms specifically during developmental growth, while sparing adults [31]. Cyclopamine, produced by *Veratrum californicum* (also known as western false hellebore, the California state flower), inhibits the Wnt/ Hedgehog signaling, essential in some cancers. Why would the plant produce such a selective anti-cancer drug? Actually, the plant produces a teratogen. When pregnant sheep grazed the flower on day 14 of gestation newborn lambs had the cyclopic defect (one eye in the middle of the face), skeletal and cleft palate birth defects [32,33]. Other plant sources contain related alkaloids. Interestingly, pregnant women experience nausea and vomiting, which peak during weeks 8 to 12. Morning sickness is characterized by aversion to strong smelling vegetables [34]. This may protect the developing embryo from teratogens produced by vegetables and food associated microorganisms. Perhaps, females have evolved to sense plant xenobiotics [34]. This suggestion is analogous to the xenohormesis model.

On the other hand, a target of a xenobiotic could be essential early in life and cause aging later in life. And TOR is such a target. Its knockout is lethal during embryogenesis [35,36]. Similarly, a double knockout of S6K1 and S6K2  is lethal [37]. In yeast, deletion of both TOR genes or TOR2 only is lethal [20]. Certainly, TOR is a perfect target for an anti-fungal drug. And, in fact, soil bacteria produce rapamycin to inhibit yeast growth.  Thus, rapamycin is a mirror image of penicillin that is produced by fungi to inhibit bacterial growth.

Although not created for that purpose by nature, inhibitors of mTOR are used as anticancer agents [38]. Furthermore, rapamycin prevents cancer in humans [39], perhaps, as an ‘anti-aging side effect' [40]. Rapamycin is also indicated for the therapy of almost all age-related diseases from atherosclerosis to macular degeneration [41,42]. However, bacteria produce rapamycin not as an aging suppressant, nor as a medicine for longevity, but rather as a growth suppressant. Growth suppressants may suppress aging because aging is a continuation of growth, driven by the same nutrient sensing TOR/S6K pathway.

## Back to resveratrol

Thus, the anti-aging effect of resveratrol may be just a side effect of targeting S6K, sirtuins and AMPK. However, there are two indirect arguments about why animals might have evolved ‘target of resveratrol' for their benefit (in agreement with the xenohormesis model). First, resveratrol (unlike rapamycin) has multiple targets in the same nutrient-sensing network: sirtuins, S6K, AMPK and perhaps several others. Why so many? Second, resveratrol toxicity seems to be unrelated to these targets. So let us consider a modified (taking into account that growth and aging are linked) xenohormesis model. Plants produce resveratrol to protect grapes from parasites by affecting ‘target X' (an intended target of resveratrol). Levels of resveratrol will be raised during plant infections, heralding the possibility of famine. Animals thus evolved targets inside the TOR/sirtuin network in order to sense resveratrol. (Note: The purpose is not to suppress aging. In the wild, lifespan is not limited by aging, especially during famine.) Perhaps, animals evolved sensors for resveratrol in the growth promoting pathway in order to inhibit growth. Thus resveratrol (mimicking lack of nutrients) can restrain growth of the animal, and a smaller animal would need less food during a forthcoming famine. But, coincidentally, the inhibition of S6K also inhibits aging.

## Conclusion

Thus, there are two alternative (but not mutually exclusive) models of the ‘natural purpose' of anti-aging drugs. First, according to the xenohormesis model [28,29], animals evolved the ability to sense their environment in order to modulate their functions, stress resistance or growth. This is consistent with there being multiple targets for resveratrol exactly in the pathway that senses the environment. Second, according to the anti-aging side effect model, plants and bacteria produce growth suppressants to hurt parasites and competitors. Yet, the same growth promoting pathway is also involved in aging. In the wild, growth suppressants exclusively hurt young organisms by inhibiting their developmental growth. In the protected environment, when animals live long enough to experience aging, the aging suppressant side effect becomes apparent.
